# GUCY2C-targeted chimeric antigen receptor expressing T cells extend survival in a therapeutic mouse model of metastatic colorectal cancer

**DOI:** 10.1186/2051-1426-1-S1-P22

**Published:** 2013-11-07

**Authors:** Michael S Magee, Adam E Snook, Adam R Hersperger, Glen P Marszalowicz, Scott A Waldman

**Affiliations:** 1 Pharmacology and Experimental Therapeutics, Thomas Jefferson University, Philadelphia, PA, USA; 2 Microbiology and Immunology, Thomas Jefferson University, Philadelphia, PA, USA; 3 School of Biomedical Engineering, Science & Health Systems, Drexel University, Philadelphia, PA, USA

## 

Adoptive T cell therapy (ACT) is an emerging cancer treatment paradigm with success in early phase clinical trials in melanoma and B cell leukemia. However, ACT has been unsuccessful in tumors arising from the colorectum, in part due to antigen-dependent “on-target off-tumor” toxicities producing damage to normal tissues. These adverse events reflect the use of affinity-enhanced T cell receptors which increase the risk of T cell-mediated damage to normal tissues expressing the target antigen, an effect which is amplified for antigens broadly expressed by different tissues. In that context, we have generated an antibody-based chimeric antigen receptor (CAR) targeting the cancer mucosa antigen guanylyl cyclase C (GUCY2C), a membrane-bound cyclase selectively produced on apical surfaces of intestinal epithelial cells (IECs) in small and large intestine, whose expression is maintained by >95% of metastatic colorectal tumors. We hypothesized that anatomical compartmentalization of GUCY2C, normally limited to luminal surfaces of intestine but conserved on all colorectal cancer cells, would enable GUC2YC-targeted CAR T cells to eliminate metastatic colorectal tumors in the absence of intestinal damage. Here, CARs specific for mouse or human GUCY2C were inserted into murine CD8+ T cells by retroviral-mediated gene transfer. GUCY2C-specific CAR T cells induced GUCY2C-dependent T cell activation quantified by staining CD25 and CD69 surface markers and intracellular accumulation of IFNγ, TNFα, and MIP1α. Further, GUCY2C-specific CAR T cells lysed CT26 mouse colon cancer cells, quantified by release of β–galactosidase, in a GUCY2C-dependent fashion. Moreover, these CAR T cells extended median and overall survival in therapeutic mouse models of GUCY2C-expressing colorectal cancer metastatic to lung. Importantly, the therapeutic effects of GUCY2C-CAR T cells were not associated with antigen-dependent clinical toxicity, including diarrhea, rectal bleeding, or rectal prolapse, or gross pathology, including hemorrhage or ulceration, in intestine. Together, these observations establish proof-of-principle that adoptive T cell therapy using GUCY2C-targeted CARs is therapeutically effective, in the absence of “on-target off-tumor” normal tissue destruction, in metastatic colorectal cancer.

